# Sp1-mediated transcriptional activation of miR-205 promotes radioresistance in esophageal squamous cell carcinoma

**DOI:** 10.18632/oncotarget.13902

**Published:** 2016-12-11

**Authors:** Fei Pan, Hui Mao, Fangfang Bu, Xin Tong, Jingjing Li, Sujie Zhang, Xing Liu, Lingxiong Wang, Liangliang Wu, Rui Chen, Huafeng Wei, Bohua Li, Cheng Li, Yunsheng Yang, Clifford J. Steer, Jian Zhao, Yajun Guo

**Affiliations:** ^1^ Key Laboratory of Cancer Center, Chinese PLA General Hospital & Chinese PLA Medical School, Beijing, P.R. China; ^2^ International Joint Cancer Institute, the Second Military Medical University, Shanghai, P.R. China; ^3^ Department of Gastroenterology and Hepatology, Chinese PLA General Hospital, Beijing, P.R. China; ^4^ Beijing Key Laboratory of Cell Engineering & Antibody, Beijing, P.R. China; ^5^ The 150th Hospital of Chinese PLA, Luoyang, P.R. China; ^6^ Departments of Medicine and Genetics, Cell Biology and Development, University of Minnesota Medical School, Minneapolis, Minnesota, USA; ^7^ State Key Laboratory of Antibody Medicine and Targeting Therapy, Shanghai, P.R. China

**Keywords:** miR-205, Sp1, radioresistance, epithelial-mesenchymal transition, esophageal squamous cell carcinoma

## Abstract

Radiotherapy for esophageal squamous cell carcinoma (ESCC) patients is limited by resistance to ionizing radiation (IR). However, the roles and mechanisms of microRNAs in radioresistance are obscure. Here, we investigated that microRNA-205 (miR-205) was upregulated in radioresistant (RR) ESCC cells compared with the parental cells. Overexpression of miR-205 promoted colony survival post-IR, whereas depletion of miR-205 sensitized ESCC cells to IR *in vitro* and *in vivo*. Further, we demonstrated that miR-205 promoted radioresistance by enhancing DNA repair, inhibiting apoptosis and activating epithelial-mesenchymal transition (EMT). Mechanistically, miR-205, upregulated post-IR, was demonstrated to be activated by Sp1 in parallel with its host gene, miR-205HG, both of which showed a perfect correlation. We also identified and validated phosphatase and tensin homolog (PTEN), as a target of miR-205 that promoted radioresistance via PI3K/AKT pathway. Lastly, increased miR-205 expression was closely associated with decreased PTEN expression in ESCC tissues and miR-205 expression predicted poor prognosis in patients with ESCC. Taken together, these findings identify miR-205 as a critical determinant of radioresistance and a biomarker of prognosis. The Sp1-mediated transcriptional activation of miR-205 promotes radioresistance through PTEN via PI3K/AKT pathway in ESCC. Inhibition of miR-205 expression may be a new strategy for radiotherapy in ESCC.

## INTRODUCTION

Esophageal carcinoma is one of the most aggressive malignant tumors, ranking eighth in global morbidity and sixth in global cancer-related mortality among all types of cancers [1–3]. Histologically, esophageal squamous cell carcinoma (ESCC) is the predominant subtype and contributes to nearly 90% of all esophageal carcinomas in China [4]. Radiotherapy is one of the major ESCC treatments, but the overall prognosis for ESCC patients remains poor [3, 5]. The incidence of local recurrence is high, primarily due to intrinsic or acquired radioresistance [6]. Therefore, investigating the molecular mechanisms underlying radioresistance may ultimately improve therapeutic outcomes.

MicroRNAs (miRNAs), a class of small noncoding RNAs, post-transcriptionally regulate gene expression [7, 8]. Over the past decade, miRNAs have been found to be involved in diverse biological processes, such as development [9], cellular proliferation and differentiation [10]. Mounting evidence has indicated that miRNAs dysregulation plays an important role in the pathology of various diseases, including cancer. The discovery that miRNAs play a role in the cellular response to ionizing radiation (IR) is a relatively recent finding [11]. The cellular miRNA expression profile can change within minutes to hours following IR and these dysregulated miRNAs may promote or inhibit radioresistance. Mechanistically, miRNAs can regulate DNA damage response genes, and modulate DNA double-strand break (DSB) repair, cell cycle checkpoint activation and apoptosis [12, 13]. However, little is known regarding the mechanisms that control miRNAs dysregulation. miRNAs are transcribed from either their own genes or from introns. Up to 40% of miRNA genes may lie within the introns of protein and non-protein coding genes [14]. These genes are usually found in the sense orientation and thus are generally regulated together with their host genes [14]. Therefore, an investigation into miRNA host genes would help elucidate the regulatory mechanisms underlying miRNA expression. IR may modulate the activity of transcription factors, such as NF-kB, E2F and Sp1, which are known to activate transcription of miRNAs [15]. It is important to investigate whether these transcription factors engage in the regulation of IR-induced miRNA expression.

MiRNA-205 (miR-205), located on chromosome 1q32.2, is an important miRNAs that has been investigated in various tumor types [16, 17]. Multiple studies of miR-205 in epithelial tumors have showed that miR-205 appears to act as controversial roles depending on tumor types or its target genes [18, 19]. miR-205 expression is down-regulated in several types of cancers including breast [20], prostate [21] and renal cancer [22] and serves as a tumor suppressor by inhibiting tumor proliferation and invasion. Conversely, miR-205 is overexpressed in ovarian [23], lung [24] and head and neck squamous cell carcinoma [25] and acts as an oncogene by facilitating tumor initiation and proliferation. Recently, one study reveals that miR-205 is upregulated in response to IR and promotes radioresistance in nasopharyngeal carcinoma (NPC) [6]. Conversely, another study indicates that miR-205 inhibits DNA damage repair and sensitizes breast cancer cells to radiation [26]. However, the role of miR-205 in radiotherapy of ESCC and the underlying mechanism of IR-mediated regulation of miR-205 remains unknown.

In this study, miR-205 promoted radioresistance and development of an aggressive phenotype, characterized by improvement of DNA repair, inhibition of apoptosis and activation of epithelial-mesenchymal transition (EMT) through targeting PTEN and activating PI3K/AKT pathway. We also found that Sp1 could activate miR-205 expression in response to IR. Thus, we identified the important role and mechanism of miR-205 as a key regulator of radioresistance and showed that downregulation of miR-205 could sensitize ESCC cells to irradiation.

## RESULTS

### Radioresistant ESCC cells possess an aggressive phenotype

**Figure 1:**
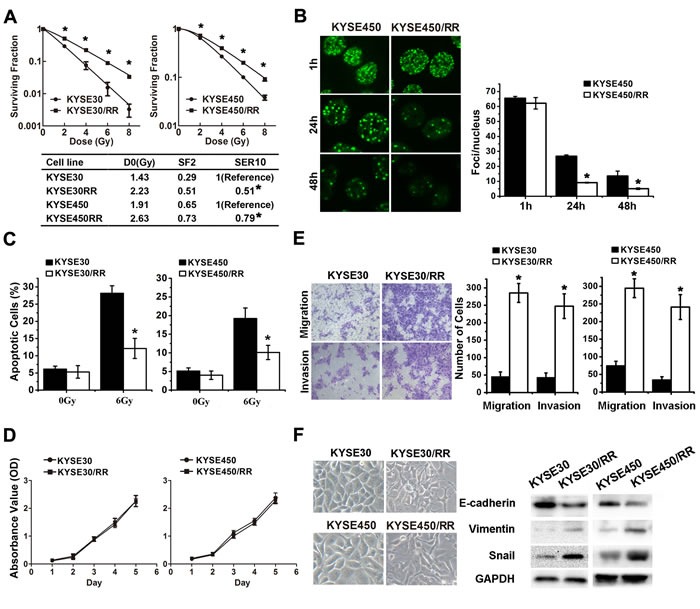
ESCC/RR cells are radiation resistant and possess an aggressive phenotype **A**. The surviving fraction of established ESCC/RR cells and their parental cells after the indicated doses of irradiation. Cancer cells were seeded into 6-well plates. Fourteen days after culturing, cells were fixed with methanol and stained with crystal violet. Colonies containing > 50 cells were counted under a microscope, and cell survival was calculated. **B**. Tumor cells were irradiated with 6 Gy and then stained with γ-H2AX antibody at the indicated times. Representative images were acquired with a confocal laser-scanning microscope. The number of γ-H2AX foci per cell was counted and calculated for 100 cells in each group. **C**. ESCC/RR cells and their parental cells were treated with or without a 6 Gy dose of IR for analysis of apoptosis. At 48 h post-IR, the cells were stained with PI and the percentage of sub-G1 cells was measured by flow cytometry. **D**. Cell proliferation assay of ESCC/RR and ESCC cells. **E**. Migration and invasion assays of ESCC/RR and ESCC cells. **F**. Morphology of ESCC/RR and ESCC cells (left); Expression levels of EMT markers (E-cadherin, Vimentin, and Snail) were analyzed by Western blots in ESCC/RR and ESCC cells (right). The data are presented as the mean ± SD of values obtained from 3 independent experiments. Statistical significance is denoted by *P < 0.05.

To simulate the clinical scenario of radioresistance, we established two radioresistant (RR) ESCC cell sublines derived from KYSE30 and KYSE450 through repeated exposure of the parental cells to IR at a dose rate of approximately 4.7 Gy/min (Supplementary Figure S1). After 10 weeks of fractionated irradiation with a total dose of 37 Gy, subclones were isolated and named KYSE30/RR and KYSE450/RR. To determine the radiosensitivity of RR cells, we performed clonogenic survival assays. KYSE30/RR and KYSE450/RR exhibited significant increases in resistance to IR compared with their parental cell lines (Figure 1A). Radiobiological parameters of the cell sublines were compared using an integrated cell survival curve, and the D0 values (the dose required to reduce survival to 37% of its original value), the SF2 (surviving fraction at 2 Gy), and the SER10 (sensitization enhancement ratio at 10%) were also determined [27].

**Table 1:** The associations of miR-205 expression with clinicopathological characteristics in ESCC patients

	Total	Low expression (n=58)	High expression (n=62)	*P* value
Age				
<60	66	36	30	0.132
>60	54	22	32	
Gender				0.800
Male	71	35	36	
Female	49	23	26	
Tumor location				0.299
Middle-Upper	75	39	36	
Lower	45	19	26	
Differentiation				0.048*
Well and Moderate	95	46	39	
Poor	25	12	23	
pT				
pT1-2	70	34	36	0.951
pT3-4	50	24	26	
pN				
pN0	21	10	11	0.943
pN1	99	48	51	
pM				
pM0	96	48	48	0.465
pM1	24	10	14	
Stage				
I-II	61	30	31	0.850
III-IV	59	28	31	

TNM, tumor, node, metastasis.

*P*-values were two-tailed and based on the Pearson χ2 test. *P < 0.05

We also characterized the phenotype of ESCC/RR cells by assessing DNA repair capacity, cell apoptosis and proliferation. First, radiation-induced DNA DSB and DNA repair kinetics were analyzed by quantifying γ-H2AX [28, 29]. The number of γ-H2AX foci in the KYSE450 cells was approximately 3 times higher than that in KYSE450/RR cells at 24-48 h after IR (Figure 1B). Subsequently, apoptosis was analyzed by flow cytometry. The fraction of apoptotic cells post-IR was decreased by 57.1% and 47.4%, in KYSE30/RR and KYSE450/RR cells, respectively, compared with the parental cells (Figure 1C). Finally, MTS assays revealed no difference in cell proliferation between the RR cells and control cells (Figure 1D).

Evidence suggests that EMT plays a crucial role in cancer radioresistance. Therefore, we further investigated the metastatic potential and EMT phenotype of RR cells. Migration and invasion assays showed that RR cells acquired a migratory and invasive phenotype (Figure 1E). Increases in cell migration (3.8-6.1-fold) and invasion (5.2-6.8-fold) were observed in the RR cells compared with the parental cells. As shown in Figure 1F left, the two RR cell lines developed a spindle-like morphology, with increased formation of pseudopodia and a loss of cell-to-cell contact. These alterations were consistent with the morphological changes of EMT, presenting decreased expression of the epithelial marker E-cadherin and increased expressions of mesenchymal markers Vimentin and Snail (Figure 1F right). Collectively, these results indicate that the ESCC/RR cells acquire a more aggressive phenotype characterized by improvement of DNA repair, inhibition of apoptosis, increased invasive potential and activation of EMT.

### miR-205 promotes radiation resistance and development of an aggressive phenotype

**Figure 2:**
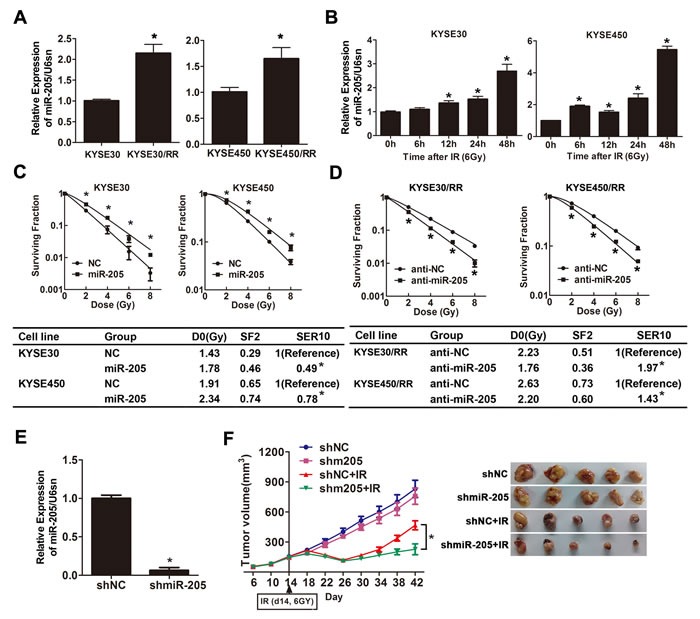
miR-205 promotes radioresistance of ESCC *in vitro* and *in vivo* **A**. miR-205 expression in ESCC/RR and ESCC cells was determined by qRT-PCR. U6sn served as an internal control. **B**. miR-205 expression was detected by qRT-PCR in three types of ESCC cell lines at the indicated time following a 6 Gy dose of IR. **C**.-**D**. ESCC cells were transfected with miR-205 agomir or agoNC as indicated. ESCC/RR cells were transfected with miR-205 antagomir or antagoNC as indicated. Twenty-four hours after transfection, cells were treated with the indicated IR dose. The results are presented as the mean ± SD of values obtained from 3 independent experiments. *P < 0.05. **E**. miR-205 expression was detected by qRT-PCR in the shmiR-205 and shNC groups. **F**. Nude mice were subcutaneously injected into the right posterior flank with 4 × 10^6^ cells infected with shmiR-205 or shNC. When the average tumor volume reached approximately 200 mm^3^, the tumors were either irradiated with a single 6 Gy dose of IR or not. The data are presented as tumor growth curves. Time to reach endpoint is shown as the mean and SEM with statistical significance denoted.

Accumulating evidence has shown that miRNAs play an important role in tumor radioresistance [13, 30] and miR-205 has been investigated to be associated with radioresistace in NPC [6] and breast cancer [26]. We thus analyzed miR-205 expression in ESCC cells in response to IR treatment. First, we compared miR-205 expression in ESCC/RR and their parental cell lines, and the results showed that miR-205 expression was increased by 2.1- and 1.6-fold in KYSE30/RR and KYSE450/RR cells, respectively (Figure 2A). Then, to examine the early effects of IR on miR-205 expression in ESCC cells, we exposed KYSE30 and KYSE450 cells to IR (6 Gy) for defined intervals. As detected by qRT-PCR, miR-205 was significantly increased in these cells as early as 6-12 h after IR (Figure 2B). The results above suggest that ESCC/RR cells show increased expression of miR-205 and that upregulation of miR-205 is an early event in response to IR.

The functional consequences of IR-induced miR-205 expression warranted further investigation. We elevated miR-205 levels by transfecting miR-205 agomir into parental cells and decreased miR-205 levels by transfecting miR-205 antagomir into RR cells. miR-205 expression was confirmed by qRT-PCR 2 to 10 days after transfection (Supplementary Figures S2-S3). Cell survival upon IR showed that miR-205 overexpression induced radioresistance in parental cells (Figure 2C), while miR-205 depletion significantly decreased the surviving fraction of RR cells post-IR (Figure 2D). Combined with the results of radiobiological parameters, these findings indicated that miR-205 promoted radioresistance *in vitro* and that decreased expression of miR-205 might possess radiosensitization potential.

To confirm the radiosensitive effect of miR-205 depletion *in vivo*, we infected KYSE450 cells with lentiviruses encoding shmiR-205 or control shRNA. miR-205 expression was detected by qRT-PCR (Figure 2E). Subsequently, KYSE450-LV-shmiR-205 and KYSE450-LV-shNon cells were inoculated into the flanks of nude mice to establish subcutaneous xenografts that were then treated with a 0 or 6 Gy dose of IR. In the absence of IR, tumor growth was slightly decreased upon miR-205 depletion. However, after exposure to IR, the KYSE450-LV-shmiR-205 tumor growth was dramatically slower than that of the control tumors. At 28 days after radiotherapy, KYSE450-LV-shmiR-205 tumor volumes were diminished by 68% *versus* 44% in KYSE450-LV-shNon tumors (*P* = 0.012) (Figure 2F). These data suggest that miR-205 depletion sensitizes ESCC cells to irradiation treatment both *in vitro* and *in vivo*.

**Figure 3:**
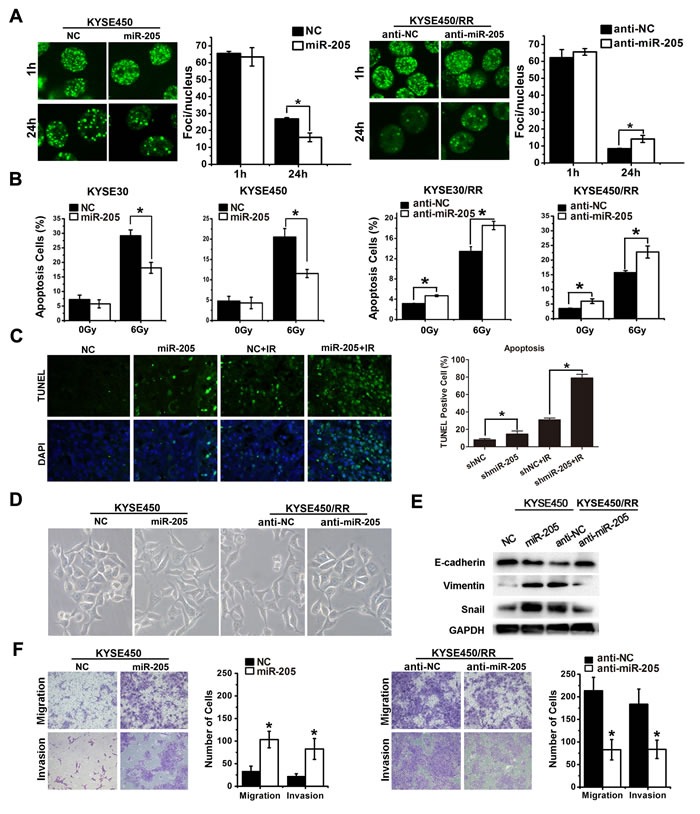
miR-205 increases radioresistance and confers an aggressive phenotype by improving DNA repair, inhibiting apoptosis and activating EMT **A**. γ-H2AX foci (a total of 100 nuclei were counted) of ESCC-NC, ESCC-miR-205, ESCC/RR-anti-NC, and ESCC/RR-anti-miR-205 cells at 1 h and 24 h following exposure to a 6 Gy dose of IR. **B**. Apoptosis was measured by flow cytometry in ESCC-NC, ESCC-miR-205, ESCC/RR-anti-NC, and ESCC/RR-anti-miR-205 cells at 48 h following exposure to a 0 or 6 Gy dose of IR. **C**. Apoptosis was measured by *in situ* TUNEL assay in KYSE450 xenografts with shmiR-205 or shNC treatment. **D**. Morphology of ESCC-NC, ESCC-miR-205, ESCC/RR-anti-NC, and ESCC/RR-anti-miR-205 cells. **E**. Expression levels of EMT markers (E-cadherin, Vimentin, and Snail) were analyzed by Western blots in ESCC-NC and ESCC-miR-205 cells. The same markers were analyzed between ESCC/RR-anti-NC and ESCC/RR-anti-miR-205 cells. **F**. Migration and invasion assays of ESCC-NC, ESCC-miR-205, ESCC/RR-anti-NC, and ESCC/RR-anti-miR-205 cells. The data are presented as the mean ± SD of values obtained from 3 independent experiments. *P < 0.05.

We next explored the mechanism by which miR-205 promoted radioresistance of tumor cells. We assessed DSB induction and repair by quantifying γ-H2AX. As shown in Figure 3A, the numbers of γ-H2AX foci were similar in all cell types at 1 h post-IR. However, a decrease in foci number was clearly observed in miR-205 agomir-transfected KYSE450 cells compared with the control cells 24 h after IR. In contrast, the number of foci in miR-205 antagomir-transfected KYSE450/RR cells was 3-5 times higher than that in the control cells 24h after IR (Figure 3A), suggesting that DSB repair was inhibited after miR-205 depletion. Apoptosis was analyzed by flow cytometry and *in situ* TUNEL assay. As shown in Figure 3B, miR-205 overexpression in KYSE30 and KYSE450 cells caused 41.4% and 43.9% decreases in apoptotic cells, respectively. In contrast, miR-205 depletion caused 37.1% and 40.6% increases in apoptotic cells in KYSE30/RR and KYSE450/RR cells, respectively. Moreover, miR-205 depletion slightly increased the apoptotic rate of KYSE30/RR and KYSE450/RR cells in the absence of IR. Consistent with the *in vitro* results, greater percentages of apoptotic cells were observed in KYSE450 xenografts with shmiR-205 treatment both with and without IR (Figure 3C). The data presented in Figure 1F showed that RR cells had undergone EMT with increased cell migration and invasion. Similarly, miR-205 overexpression in KYSE450 cells induced EMT morphologic changes (Figure 3D), accompanied with decreased expression of E-cadherin and increased expression of Vimentin and Snail (Figure 3E). Furthermore, overexpression of miR-205 in KYSE450 cells promoted cell migration and invasion, while inhibition of miR-205 in KYSE450/RR cells decreased their migratory and invasive potentials (Figure 3F). Lastly, miR-205 exhibited no significant effect on cell cycle between ESCC and ESCC/RR cells post-IR (Supplementary Figure S4). Taken together, these findings indicate that miR-205 is associated with an aggressive phenotype through numerous potential mechanisms, including improved DNA repair, inhibited apoptosis, increased invasive potential and activated EMT, similar to that observed in RR cells.

### The identification of putative transcriptional activity of miR-205 promoter

**Figure 4:**
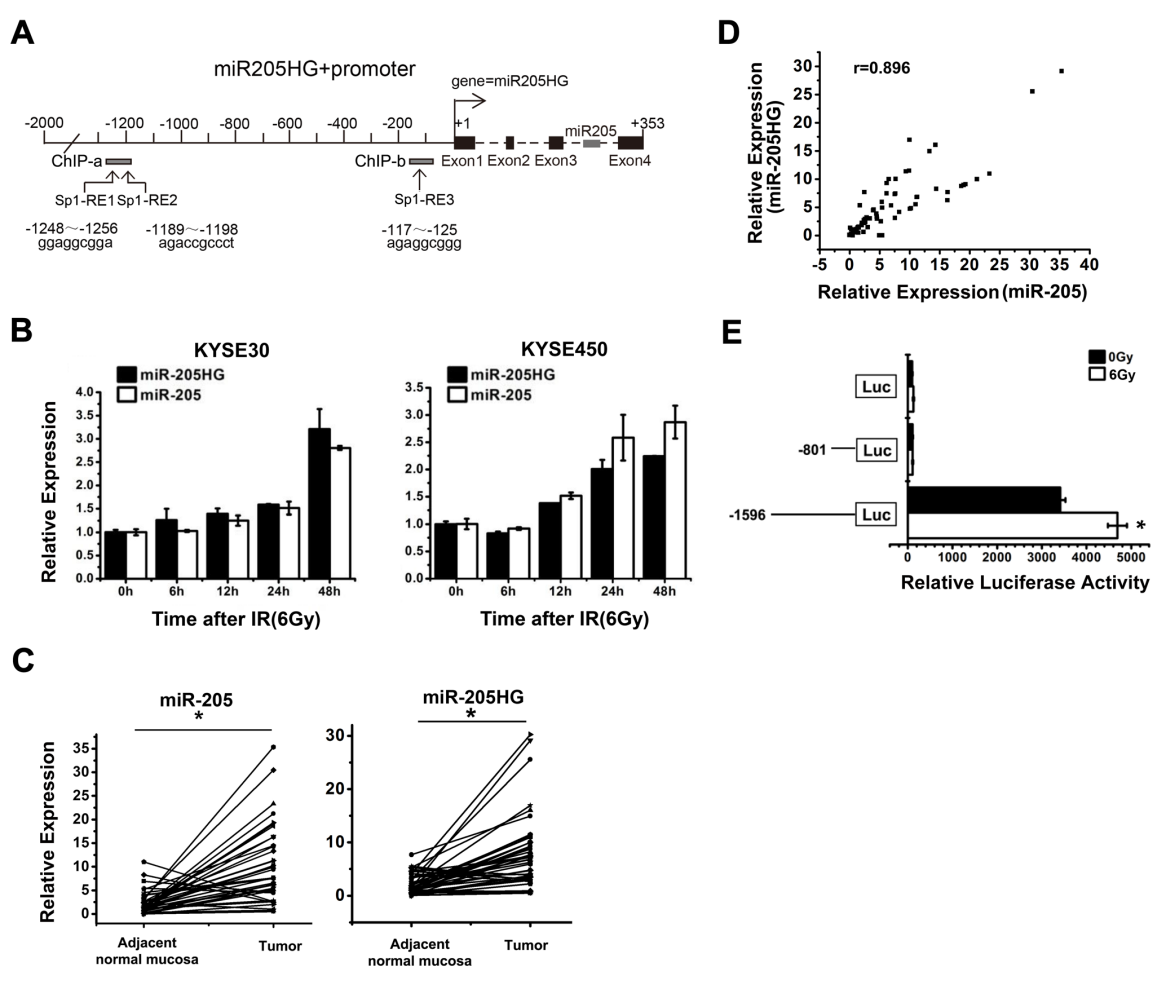
The identification of the putative transcriptional activity of miR-205 promoter **A**. MiR-205 is located within the third intron of its host gene, miR-205HG (ENST00000458250) on chromosome 1q32.2. Three putative Sp1-REs were identified: -1256/-1248 bp, -1196/-1189 bp, and -125/-117 bp upstream of the miR-205 TSS. **B**. qRT-PCR showed that miR-205HG expression, which was correlated with miR-205 expression, was increased in KYSE30 and KYSE450 cells at the indicated time following exposure to a 6 Gy dose of IR. **C**. qRT-PCR showed that both miR-205 and miR-205HG expression levels were significantly increased in ESCC tissues, compared with adjacent normal mucosa, *P* < 0.0001. **D**. miR-205HG expression showed an almost perfect correlation with miR-205 expression in ESCC tissues, Spearman's r = 0.861, *P* < 0.001. **E**. Promoter reporter assays demonstrated that the critical region for the transcriptional activity of the miR-205 promoter was located within the -1596/-801 region. The data are presented as the mean ± SD of values obtained from 3 independent experiments. *P < 0.05.

To determine the mechanism that drives miR-205 expression, we first sought to identify its primary precursor transcripts and promoter. miR-205 is located within the third intron of its host gene, miR-205HG (ENST00000458250, http://genome.ucsc.edu/), suggesting that miR-205 was co-transcribed with the miR-205HG primary RNA transcript (Figure 4A). To confirm this hypothesis, we exposed both KYSE30 and KYSE450 cells to IR (6 Gy) for defined intervals. Then, the expression of miR-205 and miR-205HG was detected by qRT-PCR. The results showed that the transcripts were co-upregulated post-IR, indicating that they were indeed transcribed under the control of the same promoter (Figure 4B). Compared with the adjacent normal mucosa, both miR-205 and miR-205HG expression levels were significantly increased in another 42 paired ESCC tissues (Figure 4C, *P* < 0.0001), showing an almost perfect correlation (Figure 4D, Spearman's r = 0.861, *P* < 0.001). To identify the miR-205 gene promoter region, we performed promoter reporter assays. We amplified two DNA fragments from the 5’ flanking region of miR-205HG gene from human genomic DNA and cloned them into the luciferase reporter vector pGL3. When these promoter reporter constructs were transfected into KYSE450 cells, the complete -1596/+189 fragment showed high transcriptional activity, but the -801/+189 fragment showed low transcriptional activity similar to that of the control vector. Further, exposure to IR significantly increased the transcriptional activity of the pGL3-1596/+189 reporter, but it didn't alter the transcriptional activity of the pGL3-801/+189 reporter (Figure 4E). Thus, the critical region for the transcriptional activity of the miR-205 promoter is located within the -1596/-801 region.

### Transcription factor Sp1 binds to and activates the miR-205 promoter upon IR exposure

**Figure 5:**
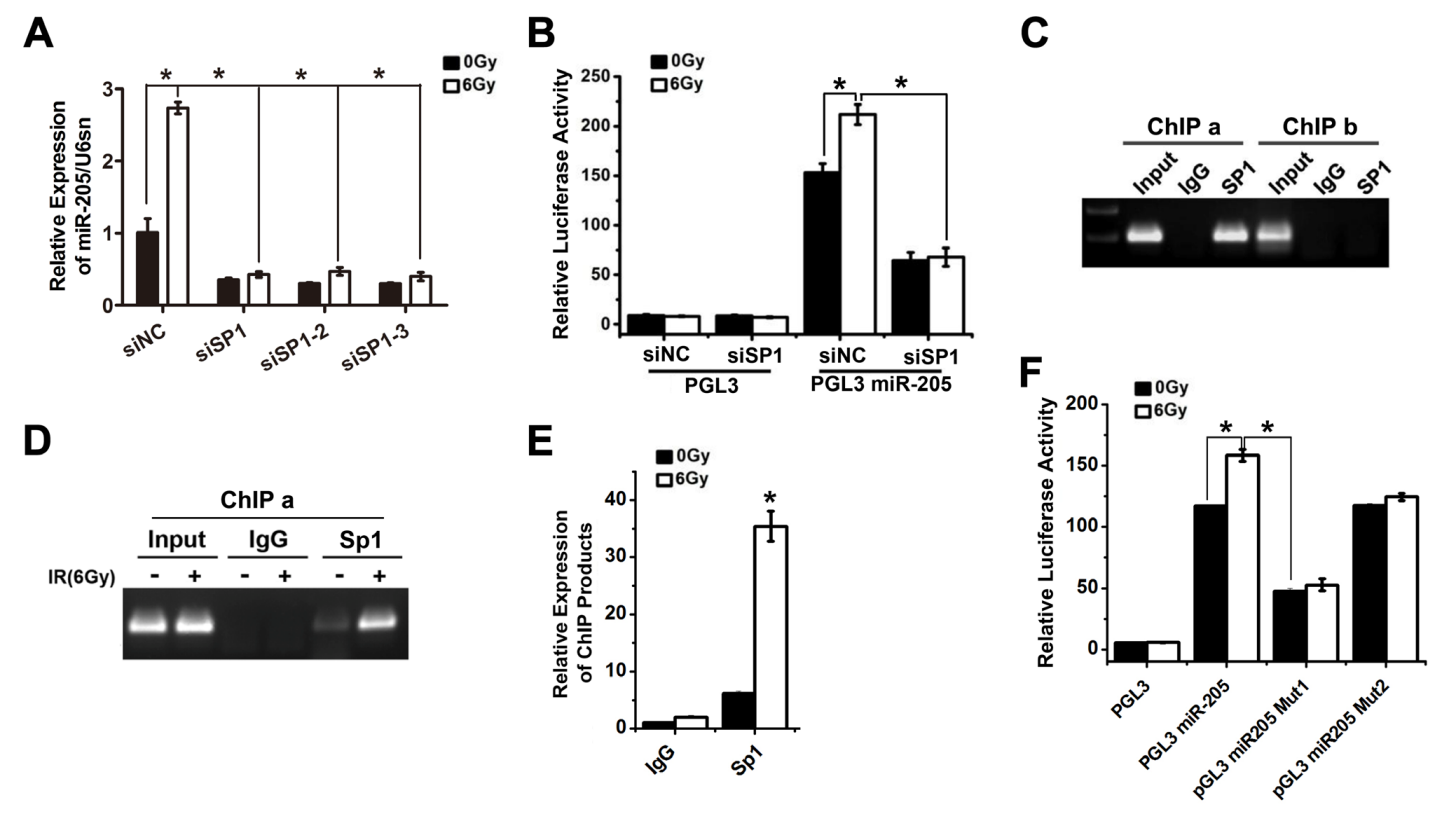
Transcription factor Sp1 binds to and activates the miR-205 promoter upon IR exposure **A**. qRT-PCR showed that silence of Sp1 decreased the basal expression level of miR-205 and abolished the IR-induced upregulation of miR-205. **B**. Luciferase reporter assays revealed that silence of Sp1 significantly reduced miR-205 promoter activities and abolished the IR-induced activation of miR-205 promoter. **C**. ChIP assays showed that Sp1 interacted with the ChIP-a region, which contained Sp1-RE1 and Sp1-RE2. Whereas, the ChIP-b region, containing Sp1-RE3, was not bound by Sp1. **D**. An additional ChIP assay showed IR could promote Sp1 binding to the ChIP-a region of miR-205 promoter. **E**. The enrichment of immunoprecipitated chromatin was monitored by qPCR. **F**. Luciferase reporter assay demonstrated that mutation of Sp1-RE1 resulted in a significant functional change compared with the wild-type promoter. The data are presented as the mean ± SD of values obtained from 3 independent experiments. *P < 0.05.

With assistance of TRANSFAC database, three putative Sp1 responsive elements (Sp1-REs) were identified (Figure 4A): Sp1-RE1, -1256/-1248 bp; Sp1-RE2, -1196/-1189 bp; Sp1-RE3, -125/-117 bp upstream of the miR-205 transcriptional start site (TSS). A previous study reported that Sp1 is phosphorylated in response to IR, which increases its transcriptional activity [15]. To verify the importance of Sp1 in miR-205 upregulation, we used three types of siRNAs to knock down Sp1 expression (Supplementary Figure S5). The qRT-PCR results showed that the three types of siRNA-induced silencing of Sp1 all decreased the basal expression of miR-205 and abolished IR-induced upregulation of miR-205 (Figure 5A). Luciferase reporter assays revealed that silence of Sp1 significantly reduced miR-205 promoter activities and abolished IR-induced activation of miR-205 promoter (Figure 5B). The results showed that Sp1 was involved in the transcriptional activation of miR-205. Chromatin immunoprecipitation (ChIP) assays were then performed to confirm Sp1 binding to miR-205 promoter. Sp1 interacted with the ChIP-a region (bp -1256 to -1189), which contained Sp1-RE1 and Sp1-RE2. Whereas, the ChIP-b region (bp -117 to -125), containing Sp1-RE3, wasn't bound by Sp1 (Figure 5C), consistent with the results shown in Figure 4E. An additional ChIP assay showed that IR promoted Sp1 binding to the cis-regulatory region (bp -1256 to -1189) of miR-205 promoter (Figure 5D). The enrichment of immunoprecipitated chromatin was also monitored by qPCR (Figure 5E). To determine the role of Sp1-RE1 and Sp1-RE2 in the transcriptional activation of miR-205, we separately generated deletion mutations of the two sites by site-directed mutagenesis. The Sp1-RE1 mutation resulted in a significant functional change compared with the wild-type promoter, suggesting a critical role of the putative Sp1-RE1 in the transcription of miR-205 (Figure 5F). The results above demonstrate that IR promotes the recruitment of Sp1 to miR-205 promoter via Sp1-RE1, thereby increasing its transcription.

### miR-205 promotes radiation resistance through down-regulation of PTEN

**Figure 6:**
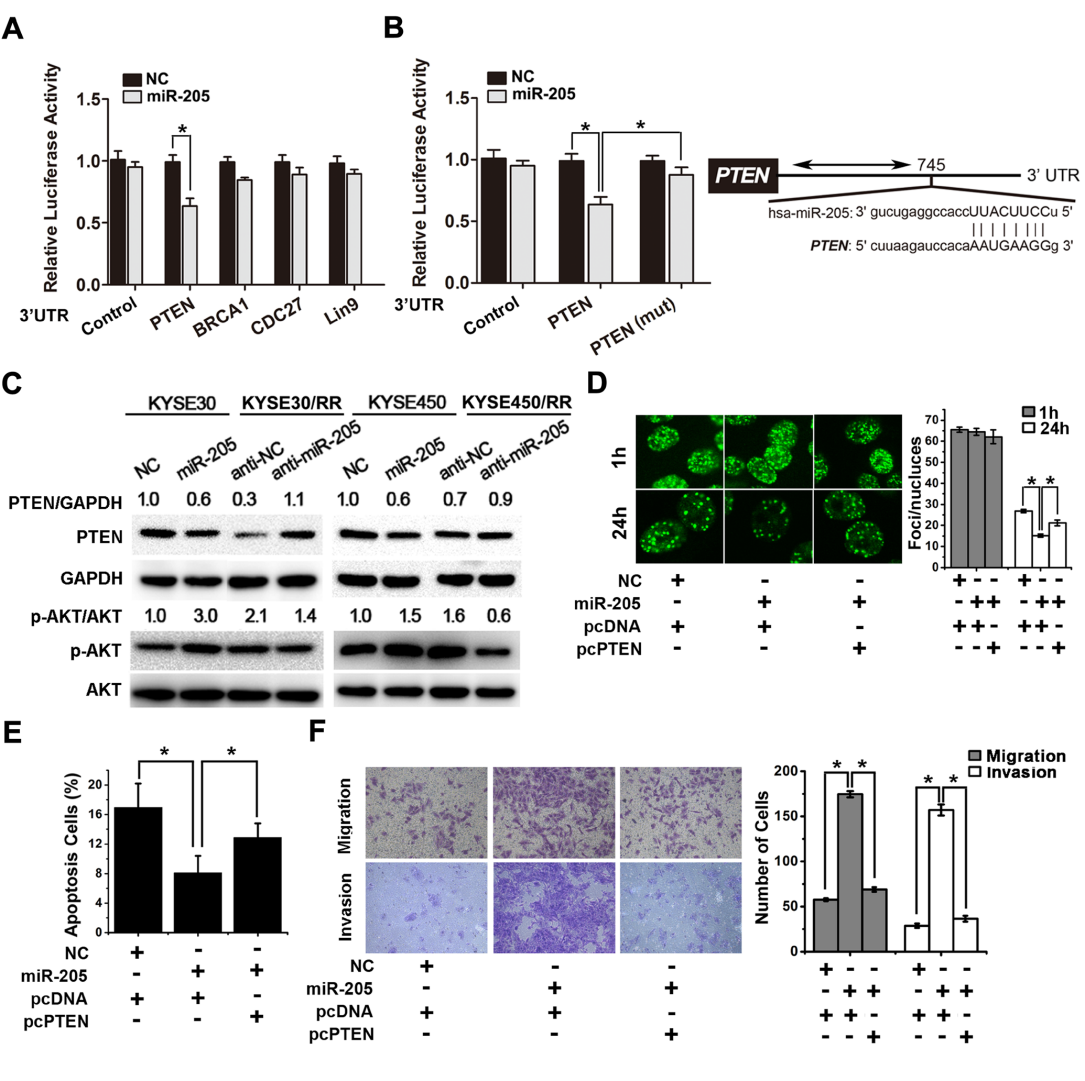
miR-205 promotes radiation resistance through down-regulation of PTEN **A**. Luciferase activity assays using luciferase reporters with wild-type human PTEN, BRCA1, LIN9 and CDC27 3′-UTR were performed after co-transfection of miR-205 mimics or NC into KYSE30 cells. The results represent the normalized ratio of firefly to *Renilla* luciferase activities. **B**. Wild type or mutant (mt) human PTEN 3′-UTR was transiently co-transfected into KYSE30 cells with luciferase reporters. The results represent the normalized ratio of firefly to *Renilla* luciferase activities. The predicted miR-205 binding site in the human PTEN 3′-UTR is shown. The data are presented as the mean ± SD of values obtained from 3 independent experiments. *P < 0.05. **C**. Western blots for PTEN, p-AKT, total AKT, and GAPDH in ESCC cells transiently transfected with miR-205 agomir or control. Western blots for PTEN, p-AKT, total AKT, and GAPDH in ESCC/RR cells transiently transfected with miR-205 antagomir or control. **D**. KYSE450 cells were transfected with miR-205, NC, pcDNA or pcPTEN, as indicated, followed by a 6 Gy dose of IR. At 1 h and 24 h post-IR, the cells were stained with γ-H2AX antibody. Representative images were acquired with a confocal laser-scanning microscope. The number of γ-H2AX foci per cell was counted and calculated for 100 cells in each group. **E**. KYSE450 cells were transfected with miR-205, NC, pcDNA, or pcPTEN, as indicated, followed by a 6 Gy dose of IR. Forty-eight hours after IR, apoptosis was measured by flow cytometry. **F**. Migration and invasion assays were performed in KYSE450 cells transfected with miR-205, NC, pcDNA or pcPTEN, as indicated.

To determine how miR-205 increases tumor radioresistance, we utilized two prediction algorithms, TargetScan 4.1 and miRanda, to analyze possible target genes. We found 202 common target genes of miR-205 (Supplementary Table S1). Efforts were focused on the identification of candidate genes that could mediate the radioresistant effects of miR-205. Several candidates were examined, including PTEN, BRCA1, LIN9 and CDC27. Candidate targets were first validated with 3′-UTR luciferase reporter assays. In miR-205 transfected cells, the luciferase reporter fused with PTEN 3′-UTR showed significantly decreased activity compared with that of the negative control (Figure 6A). In contrast, the engineered deletion mutation of the miR-205 binding site antagonized this effect (Figure 6B). These results indicate that miR-205 can directly bind to the 3′-UTR of PTEN.

We next examined whether the PTEN expression was suppressed by miR-205. As shown in Figure 6C upper, compared with the parental cells, RR cells showed a clear attenuation of PTEN protein expression, which was negatively correlated with miR-205 expression. Furthermore, overexpression of miR-205 decreased PTEN expression in parental cells, whereas inhibition of miR-205 expression significantly restored PTEN protein expression in RR cells (Figure 6C upper and Supplementary Figure S6A). Since activation of the PI3K/AKT pathway is critical for radioresistance and PTEN inhibits AKT signaling by dephosphorylating PI3K, we then examined the activity of AKT. As shown in Figure 6C lower, miR-205 overexpression increased the phosphorylation of AKT at serine 473 in parental cells, while inhibition of miR-205 expression significantly reduced AKT activity in RR cells. We also noted that PTEN overexpression decreased DNA repair in miR-205-overexpressed cells (Figure 6D and Supplementary Figure S6B) and promoted IR-induced cell apoptosis (Figure 6E). Furthermore, PTEN overexpression partially reversed the miR-205-induced increases in migration and invasion (Figure 6F). In conclusion, miR-205 promotes radioresistance by targeting PTEN *via* the activation of PI3K/AKT pathway.

### Increased miR-205 expression is associated with decreased PTEN expression in ESCC tissues and predicts poor prognosis in ESCC patients

**Figure 7:**
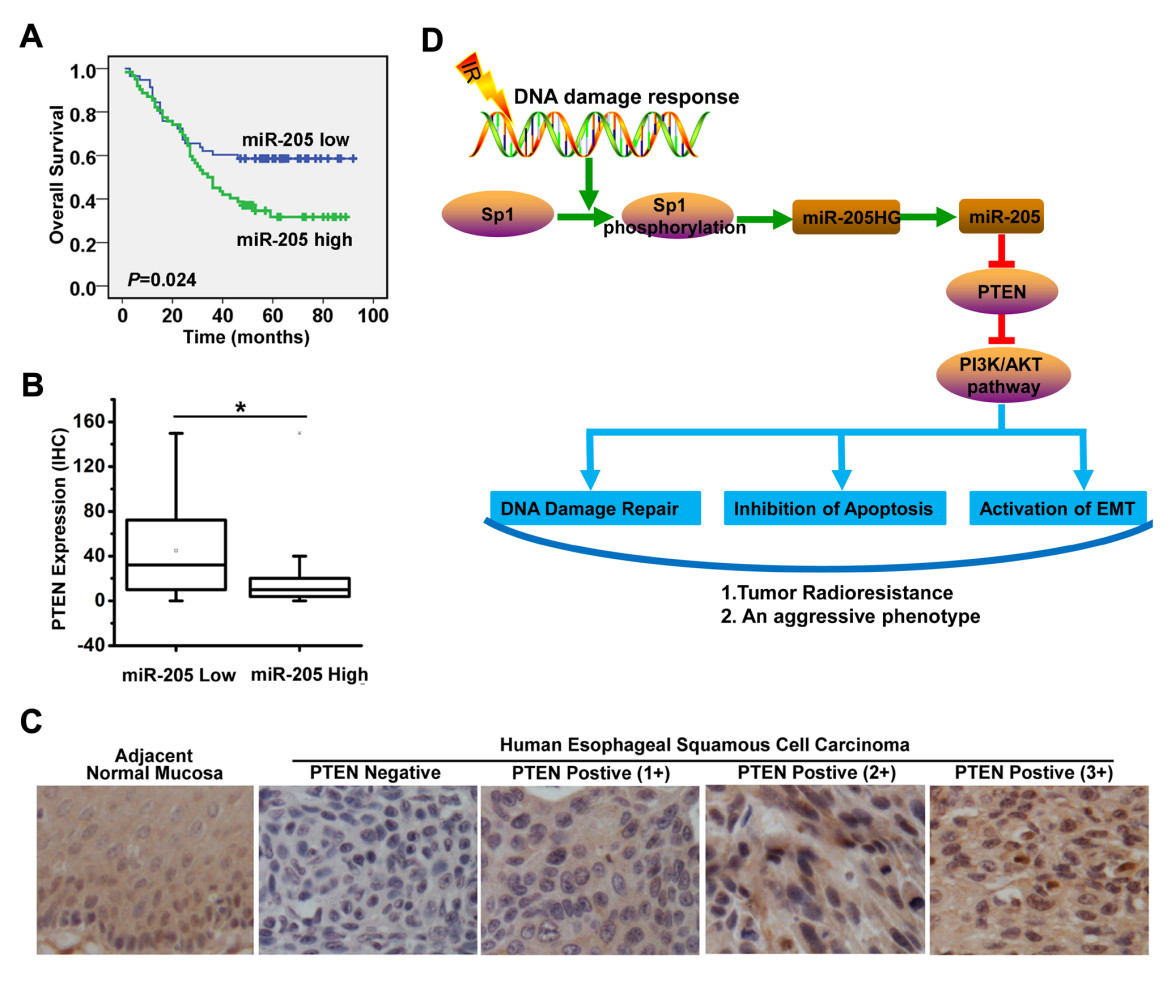
Increased miR-205 expression is associated with decreased PTEN expression in ESCC tissues and predicts poor prognosis in ESCC patients **A**. Log-rank test showed that ESCC patients with high miR-205 expression were characterized by OS compared with patients with low miR-205 expression (median survival time, 35 months *vs*. 53 months; *P* = 0.024, *n* = 120). **B**. The miR-205-low tumors showed higher scores for PTEN expression (score values: median, 32 [range, 10-76]) than the miR-205-high tumors (median, 10 [range, 4-20]); *P* < 0.001, Mann-Whitney U test, *n* = 120. **C**. Representative examples of PTEN immunostaining of tumors. **D**. The molecular mechanism underlying increased miR-205 expression and its role in radioresistance of ESCC. Radiation could phosphorylate Sp1, which increases the recruitment of Sp1 to the promoter of miR-205HG and miR-205, enhances the transcription of miR-205HG and miR-205, ultimately leading to the upregulation of miR-205 in ESCC. The Sp1-mediated transcriptional activation of miR-205 promotes radioresistance and development of an aggressive phenotype through PTEN *via* PI3K/AKT pathway in ESCC by improving DNA repair, inhibiting apoptosis and activating EMT.

To further evaluate the clinical value of miR-205 expression in human ESCC, we measured miR-205 expression in 120 formalin-fixed paraffin-embedded ESCC specimens. According to the median value of miR-205 expression in tumor tissues, 120 patients with ESCC were divided into two groups: the high-expression group (*n* = 62) and the low-expression group (*n* = 58). Log-rank test showed that ESCC patients with high miR-205 expression had poorer overall survival (OS) than patients with low miR-205 expression (median survival time, 35 months vs. 53 months; *P* = 0.024; Figure 7A). In addition, to investigate the relationship between PTEN and miR-205 expression, we measured PTEN expression by immunohistochemistry (IHC) in serial sections of human ESCC specimens (*n* = 65). These miR-205-low tumors showed higher scores for PTEN expression (score values: median, 32 [range, 10-76]) than the miR-205-high tumors (median, 10 [range, 4-20]) (*P* < 0.001, Mann-Whitney U test, Figure 7B). Representative examples of PTEN immunostaining in tumors were shown in Figure 7C. The results indicated that increased miR-205 expression was associated with decreased PTEN expression in ESCC tissues.

The associations of miR-205 expression with clinicopathological features were further analyzed. miR-205 expression level was positively associated with tumor differentiation, but was not significantly related to age, gender, tumor location, tumor size, lymph node metastasis, distant metastasis or advanced stage (Table 1). These observations prompted us to evaluate miR-205 as an independent prognostic factor of ESCC. By univariate analysis, tumor differentiation and miR-205 high expression were prognostic factors for OS. Furthermore, multivariate analysis showed that miR-205 upregulation and tumor differentiation were the two independent prognostic predictors for ESCC patients enrolled in this study (Table 2). Statistical analysis showed that miR-205 expression was significantly associated with OS in patients (hazard ratio, 1.752; 95% CI, 1.056-2.905; *P* = 0.03). Thus, the increased expression of miR-205 may serve as a prognostic indicator for patients with ESCC.

**Table 2:** Univariate and multivariate analyses of factors associated with overall survival in patients with ESCC

Variables	Hazard ration	95% CI	*P*
**Univariate analysis**			
Age (<median vs. ≥median)	0.632	0.382-1.046	0.075
Gener (female vs. male)	0.672	0.402-1.125	0.13
Differentiation (well and Moderate vs. poor)	1.921	1.112-3.318	0.019*
Location (Middle-Upper vs. Lower)	1.171	0.713-1.925	0.532
pT factor (T_1-2_ vs. T_3-4_)	1.491	0.916-2.426	0.108
pN factor (pN_0_ vs.pN_1_)	0.959	0.501-1.835	0.9
pM factor(pM_0_ vs.pM_1_)	1.177	0.661-2.097	0.579
TNM stage (I-IIvs.III -IV)	1.573	0.962-2.572	0.071
miR-205 expression (low vs. high)	1.766	1.065-2.926	0.027*
**Multivariate analysis**			
miR-205 expression (low vs. high)	1.752	1.056-2.905	0.03*
Differentiation (well and moderate vs. poor)	1.902	1.100-3.288	0.021*

Multivariate analysis, Cox proportional hazards regression model. Variables were adopted for their prognostic significance by univariate analysis and no obvious correlation between each other.

TNM, tumor, node, metastasis. *P < 0.05

## DISCUSSION

Tumor intrinsic or acquired radioresistance remains a major clinical problem [6] and little is known about the mechanisms of tumor radioresistance. To address this problem, we established two radiation-resistant cancer cell phenotypes that displayed increased survival post-IR, compared with their parental cells, which is consistent with our previous study [31]. Further, RR cells showed an increase in DNA repair capacity and a decrease in apoptosis. Recent studies have revealed that, aside from DNA damage repair [32, 33], cell cycle checkpoint [34], apoptosis [35] and autophagy [27], other biological processes including migration, invasion [36] and EMT [37] were also involved in the regulation of tumor radioresistance. Our results also testified that KYSE30/RR and KYSE450/RR cells displayed molecular and morphologic changes of EMT, accompanied with increases in cell migration and invasion.

Accumulating evidence has shown that the cellular miRNA expression profile can be notably altered post-IR [12] and that there is a strong relationship between dysregulated miRNAs and tumor sensitivity to radiotherapy [13]. Our results showed that increased expression of miR-205 was specifically found in the ESCC/RR cells and ESCC cells post-IR. As expected, elevated expression of miR-205 increased cell survival, improved DNA damage repair, and inhibited apoptosis following radiation, thus remarkably promoting radioresistance *in vitro*. In contrast, depletion of miR-205 sensitized ESCC cells to irradiation *in vitro* and *in vivo*. Further, we demonstrated that miR-205 could promote radioresistance through several biological processes, including the improvement of DNA repair, inhibition of apoptosis and activation of EMT. Our results were consistent with the findings regarding miR-205 in NPC [6], however, another study in breast cancer showed that miR-205 promoted radiosensitivity by targeting ZEB1 and Ubc13 [26]. Whether miR-205 promotes [26] or inhibits [6] radioresistance remains controversial and may be determined by the specific cancer type [16]. We identified target genes of miR-205 by TargetScan and miRanda and confirmed that PTEN was a target of miR-205 in ESCC. PTEN is a negative regulator of PI3K/AKT pathway, thereby being involved in the regulation of apoptosis, DNA damage repair and EMT during embryonic development, cancer progression and radiotherapy [38]. Our results suggested that miR-205 promoted radioresistance by inhibiting PTEN, thus activating PI3K/AKT pathway (Figure 7D). Consistent with the results regarding the miR-205/PTEN/ PI3K/AKT pathway *in vitro*, miR-205 expression was upregulated in ESCC tissues compared with adjacent noncancerous tissues and was inversely correlated with PTEN expression. Importantly, high miR-205 expression was significantly associated with poor survival of ESCC patients and could potentially serve as a prognostic indicator for patients with ESCC.

Currently, miRNA dysregulation is often reported as a driver of chemoresistance [39, 40] and radioresistance [13]. However, the mechanism driving this dysregulation remains largely unknown. Different regulatory mechanisms can control miRNA expression at genetic or epigenetic levels and may involve the biogenesis machinery or recruitment of specific transcription factors [41]. miRNAs are primarily transcribed by RNA polymerase II from their own promoter or from the promoter of host gene in which they reside [42, 43]. With bioinformatics analysis, miR-205 was identified to be located within miR-205HG. Interestingly, miR-205HG expression was strongly correlated with miR-205 expression in both human ESCC tissues and cells, confirming that the two transcripts were transcribed under the control of the same promoter. With the help of TRANSFAC database, three putative Sp1 responsive elements were identified. Sp1, a major transcription factor, is involved in the transcription of multiple genes. Recently, several studies have reported that radiation can phosphorylate Sp1, which increases its transcriptional activity [15]. In human cell lines exposed to IR, Sp1 DNA-binding activity is increased in a transient and reversible manner [44]. Correspondingly, Sp1 DNA-binding is elevated in response to oxidative stress [45]. In the present study, both luciferase reporter assays and ChIP assays demonstrated that IR exposure increased the recruitment of Sp1 to the miR-205 promoter and enhanced its transcription, ultimately upregulating miR-205 and promoting radioresistance in ESCC (Figure 7D).

Based on our findings, we hypothesized that miR-205 overexpression might be an important marker of radioresistance in ESCC and evaluation of miR-205 expression might contribute to predicting possible radioresistance for ESCC patients before radiotherapy. This may find clinical application in the future.

In conclusion, our research identifies miR-205 as a potential important biomarker of prognosis for ESCC patients and a critical determinant of radioresistance. We elucidates the mechanisms of the Sp1-mediated upregulation of miR-205 in response to IR and demonstrates the ability of miR-205 to promote radioresistance through PTEN via PI3K/AKT pathway in ESCC. Inhibition of miR-205 may be the attractive target for new therapeutic strategy in radiotherapy for ESCC patients.

## MATERIALS AND METHODS

### Patients and tissue specimens

Forty-two paired fresh tissue samples, consisting of ESCC and adjacent histologically normal tissue, were procured from surgical resection specimens collected by Department of Thoracic Surgery, the 150th Hospital of Chinese PLA, Luoyang, from 2012 to 2013. Primary tumor regions and the corresponding normal tissues were stored at -80°C. None of the patients received treatment prior to surgery. The paraffin-embedded ESCC specimens used for small RNA extraction and IHC staining were isolated from 120 patients with ESCC who had undergone surgery at the same hospital between 2001 and 2005. The study was reviewed and approved by the 150th Hospital of Chinese PLA Ethics Committee. Informed consents were obtained from patients for research purposes. The expression of PTEN protein in the specimens was detected and evaluated by IHC using previously described methods [46]. Patient characteristics are provided in Table 1.

### Cell lines and ionizing radiation

ESCC cell lines (KYSE30 and KYSE450) were cultured at 37°C in an atmosphere containing 5% CO2 in RPMI 1640 supplemented with 10% fetal bovine serum. KYSE30 and KYSE450 cell lines were authenticated with short tandem repeat profiling in 2013 by Beijing Microread Gene Technology Co, Ltd [46]. ESCC cells were exposed to various doses of irradiation using the high-energy linear accelerator MBR-152OR-3 (Hitachi, Japan) at a rate of 4.73 Gy/min.

### Transfection of miRNA agomir, antagomir and siRNA

The miR-205 agomir, antagomir, mimics and inhibitors as well as siRNAs against Sp1 (Supplementary Table S2) were synthesized by GenePharma (Shanghai, China). Oligonucleotide transfection was conducted with ScreenFect®A transfection reagent (Incella, Germany) according to the manufacturer's instructions. The transfection efficiencies of cell lines used in functional studies are shown in Supplementary Figures S2-S3.

### RNA extraction and reverse transcription PCR

Total RNA was extracted from ESCC cells lines and the 40 paired frozen ESCC tissues using TRIzol reagent (Invitrogen, USA). Small RNA was extracted from the 122 ESCC paraffin specimens using the High Pure miRNA Isolation Kit (Roche, Germany) according to the manufacturer's instructions. For detection of miR-205 expression, RNA was reversely transcribed using a specific reverse-transcription primer. Then, miR-205 expression was quantified using TaqMan miRNA assays (Applied Biosystems, USA) and normalized to U6sn RNA. All procedures were performed in triplicate. The primer sequences are listed in Supplementary Table S3.

### Western blotting

Total cell lysates were prepared in 1 × SDS buffer. Equal amounts of proteins were separated by SDS-PAGE and transferred onto PDVF membranes following previously described methods [47]. After the membranes were probed with individual antibodies, antigen-antibody complex was visualized with enhanced Supersignal Chemiluminescent reagents (Pierce Biotechnology, USA) using the FluorChem E System (Protein Simple, USA). The primary antibodies against E-cadherin, Vimentin, Snail, PTEN, AKT and p-S473-AKT were purchased from Cell Signaling Technology (Beverly, USA); the Sp1 antibody was purchased from Santa Cruz Biotechnology (Santa Cruz, USA); and the GAPDH antibody was obtained from Kangcheng Biotechnology Company (Shanghai, China).

### Radiation clonogenic survival assay

Cells in exponential growth phase were seeded into six-well plates at 500, 1000, 2000, 3000, and 4000 cells per well in triplicate. After twenty-four hours, the cells were treated with a single dose of radiation (0, 2, 4, 6 or 8 Gy, respectively). Then, the cells were incubated at 37°C in a humidified 5% CO_2_ atmosphere to allow for colony formation. After incubation for 10 days, the colonies were fixed with methanol and stained with crystal violet. Colonies containing > 50 cells were counted under a dissecting microscope. A survival curve was derived by fitting the data into the following multi-target single hit model: SF = 1-(1-e^-D/D0^)^N^ using GraphPad Prism 5.0 (GraphPad Software Inc, USA). D0 values (the dose required to reduce survival to 37% of its original value). N (the extrapolation number used to measure the width of shoulder of the survival curve).

### Cell proliferation assay

Cells were seeded into 96-well culture plates (3 × 10^3^ cells/well). Cell proliferation was measured every day for 5 days using CellTiter 96 AQueous One Solution Cell Reagent (Promega, USA) according to the manufacturer's protocol. The absorbance values of each well were detected with a universal microplate reader at the wavelength of 490 nm. All experiments were performed in triplicate. The cell proliferation curve was plotted with the absorbance at each time point.

### Migration and invasion assays

Transwell migration assays were quantified *in vitro* using transwell chambers with polycarbonate membrane filters (8 μm pore size; Corning, USA) according to the manufacturer's instructions. In brief, the lower chamber was filled with 0.6 ml medium containing 20% fetal bovine serum and 0.2 ml of medium containing 3×10^5^ cells in serum-starved conditions was plated in the upper chamber and incubated at 37°C for 48 h. Then, cells that had not migrated were removed from the upper face of the filters using cotton swabs. The cells that had migrated through the membrane and attached to the bottom of the membrane were fixed and stained with crystal violet. Images of five random fields were captured from each membrane. The number of migratory cells was counted, and the extent of migration was expressed as the average number of cells per microscopic field at a magnification of 100×. The mean of triplicate assays for each experimental condition was used. Similar inserts coated with Matrigel (BD Biosciences, USA) were used to determine the invasive potential of cells by invasion assays. Two independent, blinded investigators analyzed the migration and invasion assays.

### Cell cycle analysis

Cells were treated with or without a 6 Gy dose of IR. At 24 h post-IR, the cells were trypsinized, washed in PBS and fixed in ice-cold 70% ethanol overnight. Fixed cells were collected by centrifugation, washed in PBS and resuspended in 50μg/ml propidium iodide (PI) staining buffer (Beyotime, China) and 0.1 mg/ml RNase for 30 minutes at 37°C in the dark. Fluorescence signals from PI were measured at 630 nm with 5,000 events captured per sample by a FC500 flow cytometer (Beckman Coulter, USA). Finally, the cell cycle profile and sub-G1 phase were analyzed using FlowJo 7.6.1 (Tree Star Inc., OR, USA).

### Apoptosis analysis

ESCC cells were treated with or without a 6 Gy dose of IR. Forty-eight hours later, cells were processed according to the manufacturer's protocol described above for the cell cycle assay. Then, apoptotic cells were analyzed using FlowJo 7.6.1 (Tree Star Inc., OR, USA).

### TUNEL staining assay

*In situ* apoptosis assays were performed with the Fluorescein FragEL DNA Fragmentation Detection Kit (Calbiochem, Germany). The formalin-fixed paraffin sections were deparaffinized and incubated with TUNEL reaction mixture. Apoptotic cells carrying DNA labeled with FITC-dUTP were observed under a fluorescence microscope (Olympus, Japan).

### γ-H2AX foci formation assay

Cells were grown on glass chamber slides, fixed in 4% paraformaldehyde/PBS, permeabilized with 0.2% Triton X-100/PBS, and blocked with 10% goat serum in PBS. Cells were incubated with a primary antibody against γ-H2AX (1:100) (Cell Signaling Technology, USA) at 4°C overnight, and then incubated with a goat anti-rabbit Alex Fluor 488 antibody (Invitrogen, USA) and stained with DAPI (Invitrogen, USA). All matched samples were imaged (control and test) with a confocal laser-scanning microscope (Olympus FLUOVIEW FV-1000, Japan). A total of 100 nuclei were counted. All experiments were independently performed in triplicate.

### ChIP assay

ChIP assays were performed with the Chromatin Immunoprecipitation Assay Kit (Upstate, USA) according to the manufacturer's procedures. KYSE450 cells were irradiated with 0 Gy or 6 Gy of X-rays and incubated for six hours. Then, proteins were cross-linked for 10 minutes in a solution containing 1% formaldehyde and the cells were collected after cell lysis. Cell lysates were sonicated to generate chromatin fragments of 200-1000 bp. The immunocomplex was immunoprecipitated using a specific antibody, anti-Sp1 (Santa Cruz, USA) and a non-specific IgG was used as a technical control. Purified DNA was measured by PCR using two sets of specific primers (Supplementary Table S3) located in the miR-205 promoter region.

### Luciferase reporter assays

For the 3′-UTR luciferase assays, cells were seeded into 24-well plates and transiently co-transfected with 3′-UTR luciferase reporter constructs and miRNA mimics. After 48 h, the cells were processed to quantify firefly and *Renilla* luciferase activities using the dual-luciferase assay system (Promega, USA). The results are expressed as a normalized ratio of firefly to *Renilla* luciferase activities. For miR-205 promoter luciferase assays, cells were seeded into 24-well plates and co-transfected with constructs containing various-sized fragments of the miR-205 promoter and the pRL-TK-*Renilla* plasmid (Promega, USA). After 48 h, promoter activity was measured using the dual-luciferase assay system as described above.

### Plasmid constructions

The PTEN expression vector was constructed by the insertion of the human PTEN coding sequence into the KpnI and XhoI sites of the pcDNA3.0 vector (Invitrogen, USA). For generation of the miRNA reporter constructs, the 3′-UTR segments of PTEN, BRCA1, Lin-9 and CDC27, all of which contain a putative binding site for miR-205 were amplified and cloned into the XhoI and SalI sites of the PmirGLO Dual-Luciferase miRNA Target Expression Vector (Promega, USA). The deletion mutants of PTEN 3′-UTR were generated by performing site-directed deletion of the binding sequence of miR-205. For generation of the miR-205 promoter reporter constructs, two DNA fragments of the 5’ flanking region of the miR-205HG gene were cloned into the luciferase reporter vector pGL3 (Promega, USA). The primers used are listed in Supplementary Table S3.

### Generation of stable cell lines

Lentiviral particles expressing miR-205 inhibitors and control lentiviral were purchased from GenePharma (Shanghai, China). For generation of stable miR-205 knockdown cells, KYSE450 cells were infected with lentiviral particles expressing GFP-miR-205 inhibitors (shmiR-205) or the control lentiviral (shNC). The infected cells were screened with puromycin for two weeks, and single clones were isolated to generate monoclonal cell lines.

### Tumor xenograft experiments

Five-week-old male BALB/c nude mice were housed under standard conditions and cared according to the institutional guidelines for animal care. All animal experiments were approved by the Institutional Animal Care and Use Committee (IACUC) of PLA General Hospital. The mice (*n* = 20) were randomly divided into two groups: shNC (*n* = 10) and shmiR-205 (*n* = 10). Cells (4×10^6^ in 200 μL) with or without stable miR-205 knockdown were injected subcutaneously into the right posterior flank of each BALB/c nude mouse, respectively. Tumor volumes were detected by caliper measurements every 4 days and calculated by using the formula (volume = length × width^2^/2). When xenograft tumor volumes reached approximately 200 mm^3^, the shNC and shmiR-205 groups were randomly assigned to be irradiated with 0 Gy or 6 Gy. Each group (shNC, shNC+6 GY, shmiR-205, and shmiR-205+6 Gy), contained 5 mice. Mice were sacrificed when tumors reached 1.5 cm in diameter. The tumors were then removed, weighed, and collected for further experiments.

### Accession number for a recognized data repository

miR-205HG, ENST00000458250 (http://genome.ucsc.edu/).

### Statistical analysis

Data were expressed as the mean ± SD. Student's *t*-test or paired *t*-test was performed to evaluate significant difference between two groups by SPSS 13.0 (Chicago, IL, USA). Differences in clinical characteristics between patient subgroups were evaluated using Pearson's χ^2^ tests. Survival curves were calculated using the Kaplan-Meier method, and the analysis was performed with log-rank test. Univariate and multivariate analyses were based on Cox proportional hazards regression model. *P* < 0.05 was accepted as statistically significant.

## SUPPLEMENTARY MATERIALS FIGURES AND TABLES



## Abbreviations

ChIP: chromatin immunoprecipitation
DSB: DNA double-strand breaks
EMT: epithelial-mesenchymal transition
ESCC: esophageal squamous cell carcinoma
IACUC: Institutional Animal Care and Use Committee
IHC: immunohistochemistry
IR: ionizing radiation
miR-205: microRNA-205
OS: overall survival
PI: propidium iodide
PTEN: phosphatase and tensin homolog
qRT-PCR: quantitative reverse-transcription polymerase chain reaction
RR: radioresistant
SER: sensitization enhancement ratio
TNM: tumor, node, metastasis
UTR: untranslated region.
