# Supplementary material for: Sp1-mediated transcriptional activation of miR-205 promotes radioresistance in esophageal squamous cell carcinoma
Source: Oncotarget. 2016 Dec 11;8(4):5735–52. doi: 10.18632/oncotarget.13902 (PMC5351585; doi:10.18632/oncotarget.13902)
Supplement: Supplementary file 1 [file oncotarget-08-5735-s001.pdf]

Sp1-mediated transcriptional activation of miR-205 promotes radioresistance in esophageal squamous cell carcinoma

Supplementary Material

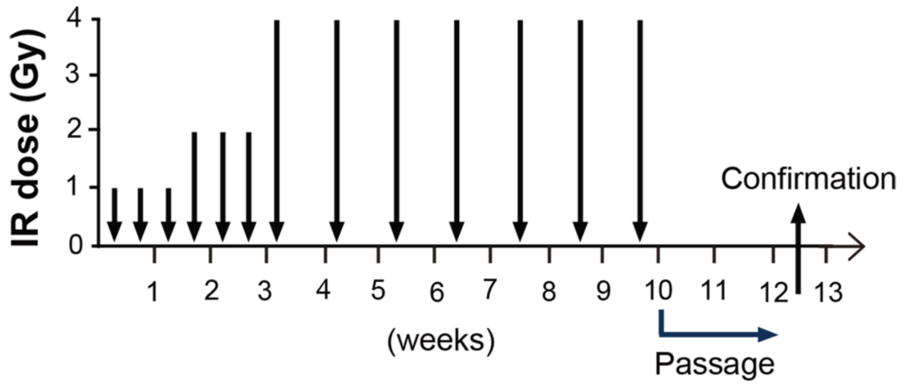

Supplementary Figure S1: Schemes for the establishment of radioresistant ESCC cell sublines.

KYSE30 and KYSE450 cells were seeded at a density of 6×10^5/T25 culture flask in complete medium. When cell confluence reached 70%, cells were treated with X-ray at an average dose rate of 4.73 Gy/min. When they reached 90% confluence, the cells were trypsinized and subcultured into new flasks. On reaching 70% confluence again, the cells were serially irradiated with increasing doses (1, 2, and 4 Gy) of radiation. These processes were repeated with 1 Gy 3 times, 2 Gy 3 times and 4 Gy 7 times to a total dose of 37 Gy for 10 weeks. Several clones were isolated from each cell line, among which, clonogenic survival assays were used to determine the resistance level. Finally, we selected a radioresistant clone from each cell line for all subsequent experiments and named them as KYSE30/RR, and KYSE450/RR.

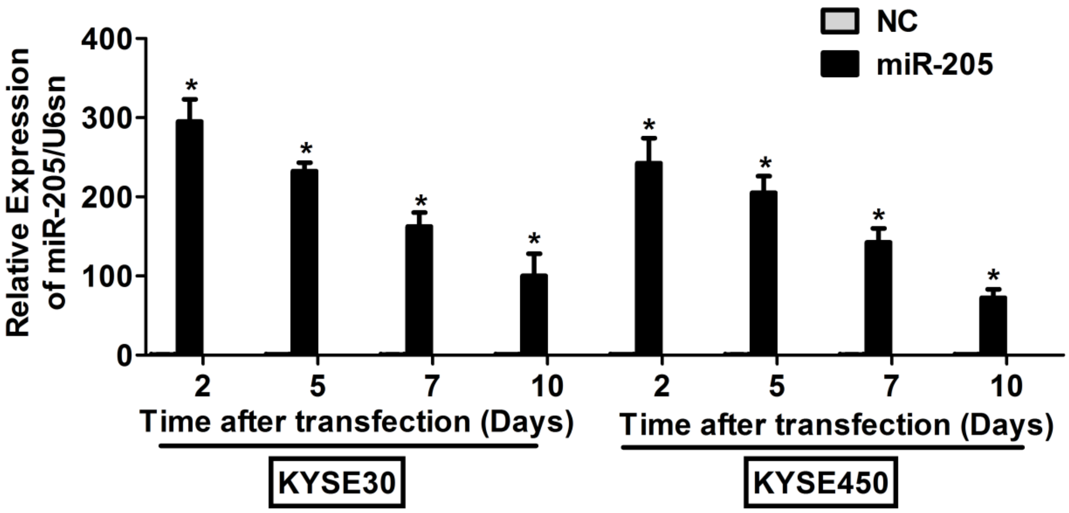

Supplementary Figure S2: Transfection efficiencies of ESCC cell lines used in functional studies.

KYSE30 and KYSE450 cells were transiently transfected with miR-205 agomir using ScreenFect®A transfection reagent. The expression of miR-205 was confirmed by qRT-PCR at different time points after transfection from 2 to 10 days. U6sn served as an internal control. The data are presented as the mean ± SD of values obtained from 3 independent experiments. *$P < 0.05$.

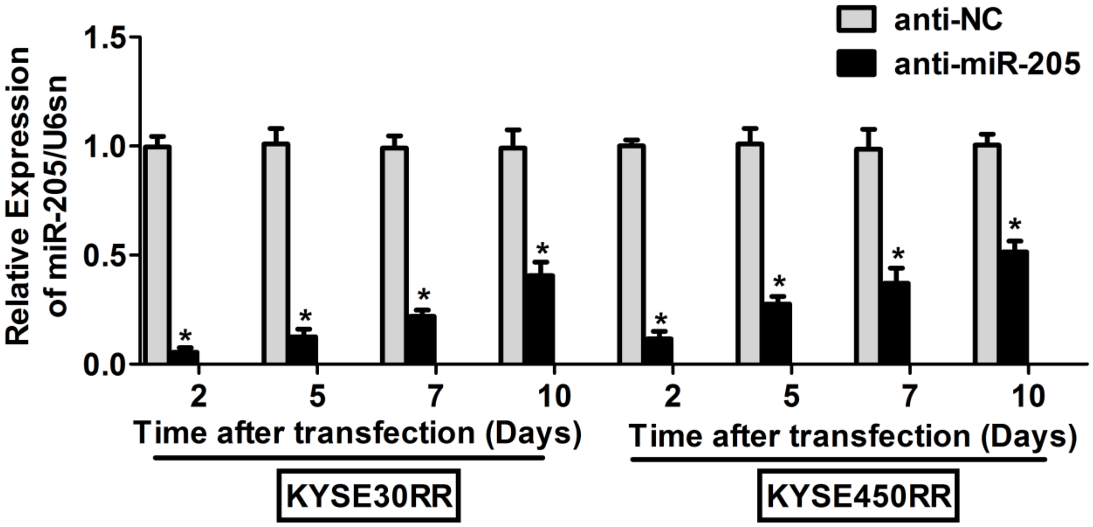

Supplementary Figure S3: Transfection efficiencies of ESCC/RR cell lines used in functional studies.

KYSE30/RR and KYSE450/RR cells were transiently transfected with miR-205 antagomir using ScreenFect®A transfection reagent. The expression of miR-205 was confirmed by qRT-PCR at different time points after transfection from 2 to 10 days. U6sn served as an internal control. The data are presented as the mean ± SD of values obtained from 3 independent experiments. *$P < 0.05$.

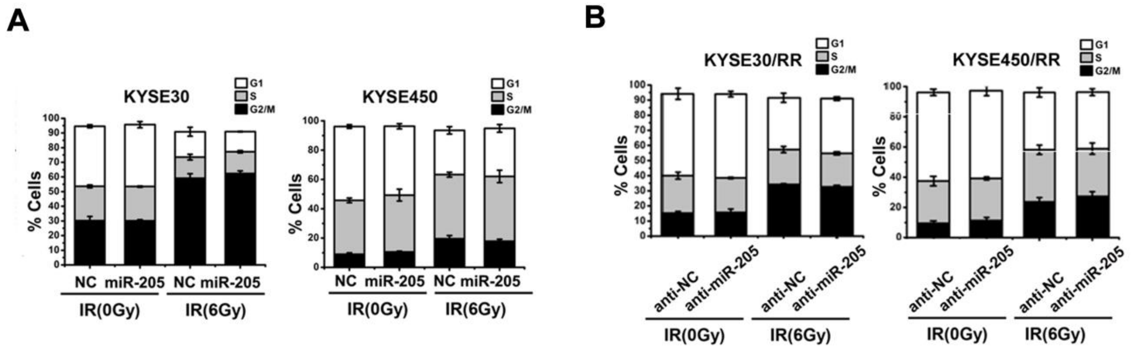

Supplementary Figure S4: The cell cycle effect of miR-205 on ESCC cells and ESCC/RR cells.

(A) The effect of miR-205 on cell cycle in KYSE30 and KYSE450 cells, as measured by PI staining. The results are presented as the mean ± SD of values obtained from three independent experiments. The data are presented as the mean ± SD of values obtained from 3 independent experiments. **P* < 0.05.

(B) The effect of miR-205 on the cell cycle in KYSE30/RR and KYSE450/RR cells, as measured by PI staining. The results are presented as the mean ± SD of values obtained from three independent experiments. The data are presented as the mean ± SD of values obtained from 3 independent experiments. **P* < 0.05.

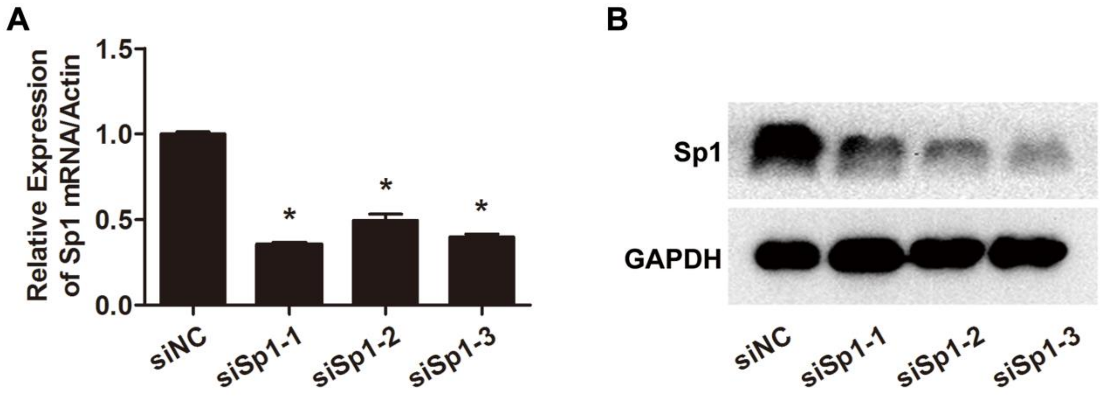

Supplementary Figure S5: Knockdown efficiencies of siRNAs against Sp1 in KYSE30 cells.

(A) KYSE30 cells were transiently transfected with three types of Sp1 siRNAs (siSp1-1, siSp1-2, siSp1-3) or a negative control (NC) using ScreenFect®A transfection reagent. Sp1 expression was measured 48 h after transfection by qRT-PCR. Student's t-tests were used to determine the statistical significance of the differences between the groups. The data are presented as the mean ± SD of values obtained from three independent experiments. Statistical significance is denoted; *$P < 0.05$.

(B) KYSE30 cells were transiently transfected with three types of Sp1 siRNAs (siSp1-1, siSp1-2, siSp1-3) or a negative control (NC) using ScreenFect®A transfection reagent. Sp1 expression was measured 48 h after transfection by Western blot.

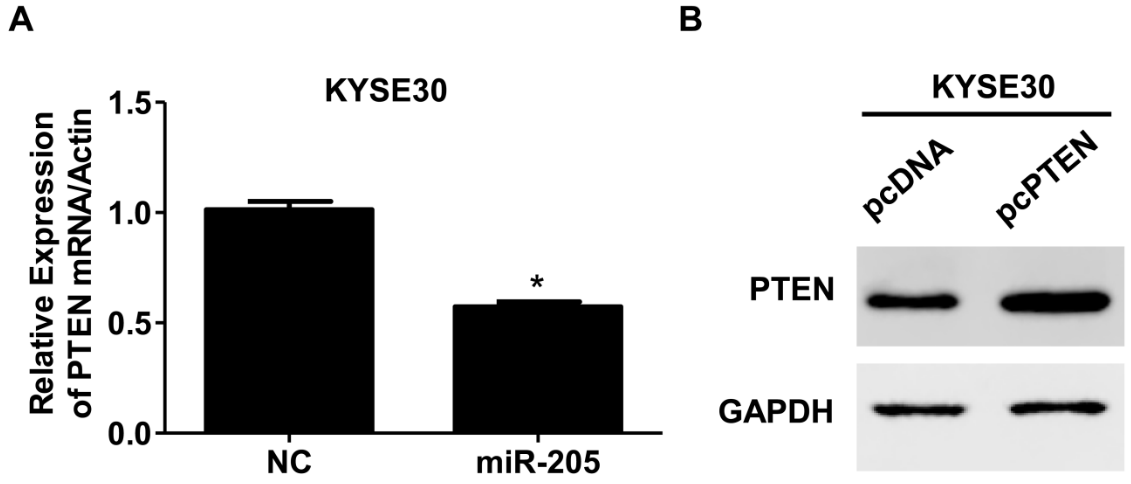

Supplementary Figure S6: PTEN expression in response to miR-205 and PTEN expression when transfected by the PTEN overexpression plasmid in KYSE30 cells.

(A) KYSE30 cells were transiently transfected with miR-205 agomir using ScreenFect®A transfection reagent using ScreenFect®A transfection reagent. PTEN expression was measured 48 h after transfection by qRT-PCR. Student's t-tests were used to determine the statistical significance of the differences between the groups. The data are presented as the mean ± SD of values obtained from three independent experiments. Statistical significance is denoted; $*P < 0.05$.

(B) KYSE30 cells were transiently transfected with pcPETN or pcDNA using ScreenFect®A transfection reagent. PTEN expression was measured 48 h after transfection by Western blot.

Supplementary Table S1. Putative target genes of miR-205

Putative Target Genes of miR-205					
ZNF606	HS3ST1	TLK1	WDR35	CASD1	CADM1
CMTM4	EFHA2	LAMC1	PTK7	**CDC27**	ENC1
DMXL2	EPB41	SUSD1	TBX3	TPP2	PJA2
BTBD3	RBPMS2	SBF2	C11orf34	MGA	CASC4
LPCAT1	DUSP7	FRK	ZNF536	UBE2N	LYSMD3
YES1	TP53BP2	BMPER	ACSL4	SLC4A4	ABI2
CHN1	SLC35A1	SEMA7A	ZEB1	TMEM26	PHC2
DLG2	MAGI2	PDE3B	FAM196A	**PTEN**	GRAMD1C
ZFYVE16	HSD17B11	MMD	BMPR1B	IVNS1ABP	SPATA13
CCNJ	C10orf131	MAGI1	DDX5	HNRNPK	OCIAD1
PTCHD1	NACC2	FBXO22	SLC19A2	LUC7L3	TRAK2
TBX18	ENPP4	INHBA	LY75	DNAJA1	SATB2
MED1	IL1R1	KY	PHF17	PTP4A1	LCA5
LRRK2	CENPF	EZR	ANGPT2	LRP6	NAA25
MGRN1	PTPRM	INPPL1	PTPRJ	**BRCA1**	CALU
KPNA1	PCDH20	CBX1	**LIN9**	KLHL15	CXorf21
ACSL1	CLTC	VASN	ESRRG	RPS6KA3	FAM108B1
PPP1R15B	FOXF1	NR3C2	JPH4	MID1IP1	C14orf43
TAPT1	SLC35B3	TSHZ3	RGS6	NCOA1	DNM1L
C11orf86	SMAD4	HSF5	C20orf111	CBLL1	PHYHIPL
CDK19	PLCB1	HIATL1	DGCR8	RAB14	CUX2
COX11	TIMM17A	DOK4	PDS5A	RBM12	AMOT
ETNK1	ZCCHC14	CSPP1	SS18	RUNX2	C12orf23
CDK14	TC2N	DCHS1	GLIS3	CENPO	PPP1R8
TNFAIP8	HERC3	ERRFI1	GATA3	NFIB	SIAH1
RBM47	LHFPL2	TM9SF3	SEPT11	SCD5	STRBP
NEU1	C21orf63	MARCKS	CLDN8	GCOM1	LCOR
CHIC1	ERBB3	UBIAD1	NKX2-3	FAM120A	STK3
TGFA	CTPS2	B4GALT6	NDUFA4	TET1	FAM176A
NSF	SORBS1	FAM155A	PAX9	EPS15	CLDN11
ADAMTS9	PRKCE	PICALM	WHSC1L1	LRPPRC	C14orf101
LRP1	CALCRL	WWC1	PLEK	CHD2	LYPD6
PHF16	VEGFA	LPAR1	HNRNPH3	MAP3K13	CANX
CPEB2	RBMS1	STS	SP4		

Supplementary Table S2. Three types of siRNAs against Sp1

Types of siRNAs
**siSp1-1 homo-658**
Sense strand 5'-rGrUrGrCrArArArCrCrArArCrArGrArUrUrArUdTdT-3'
Antisense Sequence: 5'-rArUrArArUrCrUrGrUrUrGrGrUrUrUrGrCrArCdTdT-3'
**siSp1-2 homo-1030**
Sense strand 5'-rCrCrArGrCrArArCrArUrGrGrGrArArUrUrArUdTdT-3'
Antisense Sequence: 5'-rArUrArArUrUrCrCrCrArUrGrUrUrGrCrUrGrGdTdT-3'
**siSp1-3 homo-1682**
Sense strand 5'-rGrCrCrGrUrUrGrGrCrUrArUrArGrCrArArArUdTdT-3'
Antisense Sequence: 5'-rArUrUrUrGrCrUrArUrArGrCrCrArArCrGrGrCdTdT-3'

Supplementary Table S3. Primers used in this study

Name	Primer Sequence
**Primers for qRT-PCR**	
Sp1-F	5’TGGCAGCAGTACCAATGGC3’
Sp1-R	5’CCAGGTAGTCCTGTCAGAACTT3’
PTEN-F	5’GAAAGCTTACAGTTGGGCCCTGT3’
PTEN-R	5’GCCACAGCAAAGAATGGTGATGCT3’
**Primers for qPCR to measure enrichment of immune-precipitated chromatin**	
Sp1-RE1-F	5’AGATGGGCCGAGAGATAGG3’
Sp1-RE1-R	5’AAGAGGGCGGTCTATAAACTTG3’
Sp1-RE2-F	5’CAAGTTTATAGACCGCCCTCTT3’
Sp1-RE2-R	5’AGGCTTTGTGACCTGCTT3’
**Primers for gene cloning**	
pcSp1-F	5’GGGGTACCCCGCCACCATGAGCGACCAAGATCACTCCA3’
pcSp1-R	5’CCGCTCGAGCGGTCAGAAGCCATTGCCACTGATA3’
pcPTEN-F	5’CGGGGTACCCCGGCCACCATGACAGCCATCATCAAAGAGATC3’
pcPTEN-R	5’CCGCTCGAGCGGTCAGACTTTTGTAATTTGTGTATGC3’
**Primers for promoter cloning**	
PGL-miR-205-P1-F	5’CGGGGTACCCCG CCAGTCTATGCGGTAAGAAGTT3’
PGL-miR-205-P1-R	5’CCGCTCGAGCGGTTGAAGGAGAGGGAGTAAAGGT3’
PGL-miR-205-P2-F	5’CGGGGTACCCCG AAATCTTAGCATTCCCCTCTCC3’
PGL-miR-205-P2-R	5’CCGCTCGAGCGGTCAGCACCCACCTTATCACACC3’
PGL-miR-205-P1Mut1-F	5’CGAGAGATAGGGGAGGGGCAGCCCAAGTTGCG3’
PGL-miR-205-P1Mut1-R	5’TGCCCCTCCCCTATCTCTCGGCCCATCTTGCCA3’
PGL-miR-205-P1Mut2-F	5’TGGGGTAAAATAATCAAGTTTATCTTTAAAGTTAC3’
PGL-miR-205-P1Mut2-R	5’ATAAACTTGATTATTTTACCCCACCCCCGCTTCCATG3’
**Primers for PTEN 3'UTR cloning**	
PTEN-3'UTR-F	5’CTCGAGTTTATGCTGGACTCTGG3’
PTEN-3'UTR-R	5’GTCGAC AAGCCCATTCTTTGTT3’
PTEN-3'UTR-Mut-F	5’GCAACATCTTAAGATCCACAGATATAAAAATAATG3’
PTEN-3'UTR-Mut-R	5’TGTGGATCTTAAGATGTTGCAGAAGGTTCATTC3’

Abbreviations: F, forward primer; R, reverse primer; UTR, untranslated region.
